# Association of tumor-infiltrating T lymphocytes with intestinal-type gastric cancer molecular subtypes and outcome

**DOI:** 10.1007/s00428-020-02932-3

**Published:** 2020-09-21

**Authors:** Naziha Mansuri, Eva-Maria Birkman, Vanina D. Heuser, Minnamaija Lintunen, Annika Ålgars, Jari Sundström, Raija Ristamäki, Laura Lehtinen, Olli Carpén

**Affiliations:** 1 Research Center for Cancer, Infections and Immunity, Institute of Biomedicine, University of Turku, Kiinamyllynkatu, 10 20520 Turku, Finland; 2 Department of Pathology, University of Turku and Turku University Hospital, Kiinamyllynkatu, 10 20520 Turku, Finland; 3 Department of Oncology, Turku University Hospital, Kiinamyllynkatu 4-8, 20521 Turku, Finland; 4 Medicum Research Program in Systems Oncology and HUSLAB, University of Helsinki and Helsinki University Hospital, Haartmaninkatu 3, 00014 Helsinki, Finland

**Keywords:** Gastric cancer, Molecular subtypes, Tumor infiltration, Overall survival

## Abstract

While host immune response is likely to be important for the prognosis of gastric cancer patients, detailed information on the T lymphocyte infiltration in different gastric cancer subtypes is lacking. Here, we studied the presence of CD3, CD8, and FOXP3 (Forkhead box p3) expressing T lymphocytes in a retrospective cohort of 190 intestinal gastric and gastroesophageal adenocarcinomas. The cancers represented four distinct molecular subtypes: Epstein-Barr virus–positive (EBV+), mismatch-repair-deficient (MMR-D), aberrant TP53, and the “other” subtype. The absolute numbers of CD3+, CD8+, and FOXP3+ T lymphocytes were analyzed in relation with these molecular subtypes and selected clinicopathological parameters. Overall, there was a large variation in the amount of infiltrating T lymphocyte in all molecular subtypes. Among the subtypes, EBV+ cancers differed from the other subtypes in increased lymphocyte infiltration and high CD8+/FOXP3+ ratio. While the TP53 aberrant subtype did not differ in the absolute amount of T lymphocyte, the ratio of CD8+/FOXP3+ and CD3+/FOXP3+ cells was highest in this subtype, possibly reflecting immunosuppression associated with genomic instability. Increased CD3+ and CD8+ T lymphocyte infiltrates were associated with better survival, and remained as independent prognostic factors in a multivariate analysis. This study is the first to investigate lymphocytic infiltration within four molecular subtypes of intestinal-type gastric cancer in a European cohort. The results provide an important addition to the current knowledge of T lymphocyte–dependent immune response in gastric cancer and its prognostic significance.

## Introduction

Gastric cancer is the fifth most common cancer type globally, and the second most common cause of cancer death [1]. The common treatment modalities for gastric cancer patients include surgical resection and chemotherapy. Currently, the only targeted therapy is anti-*HER2* treatment for cancers demonstrating *HER2* amplification or polysomy [2, 3]. Other treatment modalities, including immuno-oncologic treatments, are being evaluated, but it is not clear whether gastric cancer patients benefit from these treatments and on which criteria patients should be selected [4]. Unlike the traditional modalities, these therapies target the host immune system rather than the tumor. General understanding on the role of the tumor immune microenvironment is emerging, including the relationship between the host immune system and cancer. This knowledge is being used for prognostication and guiding immunotherapy, including the use of immune checkpoint inhibitors [5].

Traditionally, gastric cancer has been divided into intestinal and diffuse subtypes, based on morphological criteria [6]. Moreover, recent studies have revealed the versatile molecular background of gastric cancer. While the diffuse subtype is molecularly fairly homogenous (genomically stable), the intestinal subtype of gastric and gastroesophageal junction cancers can be further divided into different molecular subtypes. These subtypes include (1) Epstein-Barr virus (EBV)–associated cancers, (2) mismatch-repair-deficient (MMR-D) cancers, (3) cancers with TP53 aberration, and (4) cancers lacking any of the above features (“other”) [7]. The molecular subtypes are associated with different clinical features including prognosis, the TP53 mutated tumors having the worst and EBV-positive tumors the best outcome [8]. While the original molecular classification is based on genomic analysis, it is also possible to identify these subtypes by immunohistochemistry/in situ hybridization [8]. This method utilizes markers already used in clinical practice and is therefore easily adaptable to diagnostic routine.

Based on the variable immunogenic properties of gastric cancer, it is likely that the molecular subtypes elicit different levels of host immune reaction. Tumor-infiltrating lymphocytes (TILs) are considered to be a manifestation of host immune reaction against cancer cells [9]. The amount of TILs is thought to be associated with the mechanisms controlling the growth, progression, and metastasis of cancer, and they may have predictive value in the evaluation of the response to cytotoxic treatments, such as chemotherapy and radiotherapy [10]. Among these inflammatory cells, CD3 is a marker of all mature T lymphocytes, including different functional subsets. CD8 is a marker of cytotoxic T cells, considered to be critical for tumor surveillance [11], as they can recognize and kill tumor cells. FOXP3 is a marker of regulatory T lymphocytes (Tregs). Tregs are immunosuppressive and generally suppress or downregulate the induction and proliferation of effector T cells, thereby maintaining the immunological tolerance to host tissues [12]. In several cancer types, there is a strong association between the amount of tumor-infiltrating immune cells and clinical outcome. These cancer types include non-small cell lung cancer, colorectal cancer, ovarian cancer, breast cancer, and melanoma [13–17]. However, the role of immune cells in gastric cancer is less clear, especially in association with the different molecular subtypes.

In this study, we analyzed the immune infiltrates in intestinal-type gastric and gastroesophageal adenocarcinomas. We focused on the intestinal subtype, as its molecular heterogeneity allowed us to correlate the presence of CD3+, CD8+, and FOXP3+ immune cells to specific genetic and EBV-induced features. The results were analyzed in relation with the molecular subtypes and selected clinical parameters, including disease-free and overall survival.

## Materials and methods

### Patients and tumor specimens

The collection and characteristics of the study cohort have been previously reported [8]. In brief, a total number of 190 patients with intestinal-type gastric adenocarcinomas were selected out of a consecutive series of 244 patients diagnosed with adenocarcinoma of the stomach, gastroesophageal junction (GOJ), or distal esophagus at the Turku University Hospital between years 1993 and 2012. For confirmation of diagnosis and adequacy of material, all corresponding hematoxylin-eosin (H&E)–stained slides were reviewed. Tumor stage was assessed according to the current WHO Classification manual [18]. The relevant clinical information was collected from the medical records. The median follow-up time was 125 months.

The intestinal-type cancers were classified based on the following criteria: EBER in situ hybridization–positive tumors were classified as EBV-positive, tumors showing a complete loss of nuclear reactivity of at least one of the MMR markers (MLH1, MSH2, MSH6, PMS2) were classified as MMR-D, and tumors with complete loss of or strong diffuse TP53 nuclear immunoreactivity were classified as TP53 aberrant. Tumors showing none of these alterations were classified as “other” [8]. The reporting of the study has been performed following the current recommendations [19]. The study cohort characteristics are summarized in Table 1.

**Table 1:** Patient characteristics of the intestinal-type esophagogastric adenocarcinomas

Number of patients	*n* (%)
All	190
Median age at diagnosis (range)	
	74.4 (32.9–90.9)
Patient sex	
Female	68 (35.8)
Male	122 (64.2)
Site of primary tumor	
Distal esophagus	19 (10.0)
GOJ/cardia	60 (31.6)
Corpus	52 (27.4)
Antrum/pylorus	59 (31.1)
Tumor differentiation grade	
Grade 1	17 (8.9)
Grade 2	93 (48.9)
Grade 3	80 (42.1)
Stage	
I	40 (21.1)
II	79 (41.6)
III	61 (32.1)
IV	10 (5.3)
TNM stage	
T1	19 (10.0)
T2	30 (15.8)
T3	81 (42.6)
T4	60 (31.6)
N0	81 (42.6)
N1	38 (20.0)
N2	29 (15.3)
N3	17 (8.9)
Nx	25 (13.2)
M0	179 (94.2)
M1	10 (5.3)
Mx	1 (0.5)
Follow-up status	
Alive and free of disease	34 (17.9)
Alive with disease	1 (0.5)
Deceased	155 (81.6)

### Tissue microarray construction

The ngTMA (next-generation tissue microarray) construction has been described previously [20]. The TMA blocks were sectioned, stained, scanned, and uploaded into a web portal (casecenter.utu.fi) for annotation. Four individual cores (1.0 mm in diameter) were collected from each tumor, two from the central area and two from the invasive front. The cores were evaluated separately for the number of CD3+, CD8+, and FOXP3+ T lymphocytes.

### Immunohistochemistry of T lymphocyte subsets

Immunohistochemistry (IHC) reactions were performed on 4-μm paraffin sections with BenchMark XT device (Ventana/Roche). Primary antibodies were anti-CD3 (ready-to-use rabbit monoclonal antibody clone 2GV6, Ventana/Roche), anti-CD8 (rabbit monoclonal antibody clone SP57, Ventana/Roche), and anti-FOXP3 (mouse monoclonal antibody, clone 236A/E7; Abcam, Cambridge, UK at 1:200 dilution). For CD3 staining, the epitope retrieval was performed with CC1 buffer (Ventana/Roche) and the protocol used was the mild time (30 min) protocol; the antibody incubation time was 28 min at 37 °C. For CD8 staining, the epitope retrieval was performed with CC1 buffer mild time (30 min) protocol, the antibody incubation time was 32 min at 37 °C, and the ultraView amplification kit (Ventana/Roche) was used with 4-min incubation. For FOXP3, staining was performed according to the manufacturer’s instructions. Signal detection was performed with the ultraView universal DAB Detection Kit (Ventana/Roche). CD8 and CD3 membrane staining was considered positive, while identification of FOXP3-positive T lymphocytes was based on distinct nuclear expression.

### Quantitative analysis of T lymphocytes

The quantification of intratumoral T lymphocyte was carried out essentially as described earlier for colorectal cancer [21]. IHC-stained TMA slides were scanned and downloaded from the web portal (casecenter.utu.fi). Each spot in the TMA was divided into four areas of approximately 0.2 mm^2^. Images were captured using the Pannoramic Viewer software (3DHistech, Budapest, Hungary). The absolute number of positive lymphocytes for each antibody (CD3, CD8, and FOXP3) in the 0.2-mm^2^ areas was counted using the Image J software (http://rsb.info.nih.gov/ij). Only complete tissue cores with at least 20% viable tumor tissue were included in the analysis. Evaluation of the invasive front and center of the tumors were performed separately. For the statistical analysis, the mean and median lymphocyte subset counts per tumor were used. In addition, the relative ratios of different T lymphocyte subsets were calculated (CD8+/FOXP3+ and CD3+/FOXP3+). The quantitative analyses were carried out blinded of the clinicopathologic information.

### Statistical analysis

The T lymphocyte subsets were analyzed for differences among the molecular subtypes using *χ*2 test or Fisher’s exact test. Kaplan-Meier log-rank test was used for univariate survival analysis and Cox proportional hazards regression model for multivariate analysis. Variables that were statistically significant in univariate analysis were included in the multivariate analysis. Only recurrences ≥ 6 months after the time of diagnosis were considered relevant for the recurrence-free survival (RFS) which was calculated from the time of diagnosis to the time of first recurrence, death of any cause, or to the last follow-up date. Overall survival (OS) was calculated from the time of diagnosis to the time of death of any cause or the last follow-up date. Statistical analyses were performed with IBM SPSS Statistics for Windows, version 240.0 (IBM Corporation, Armonk, NY). *p* values < 0.05 were considered statistically significant.

## Results

### Distribution of the CD3, CD8, and FOXP3 T lymphocytes among intestinal-type adenocarcinomas and their association with the molecular subtypes

**Fig. 1:**
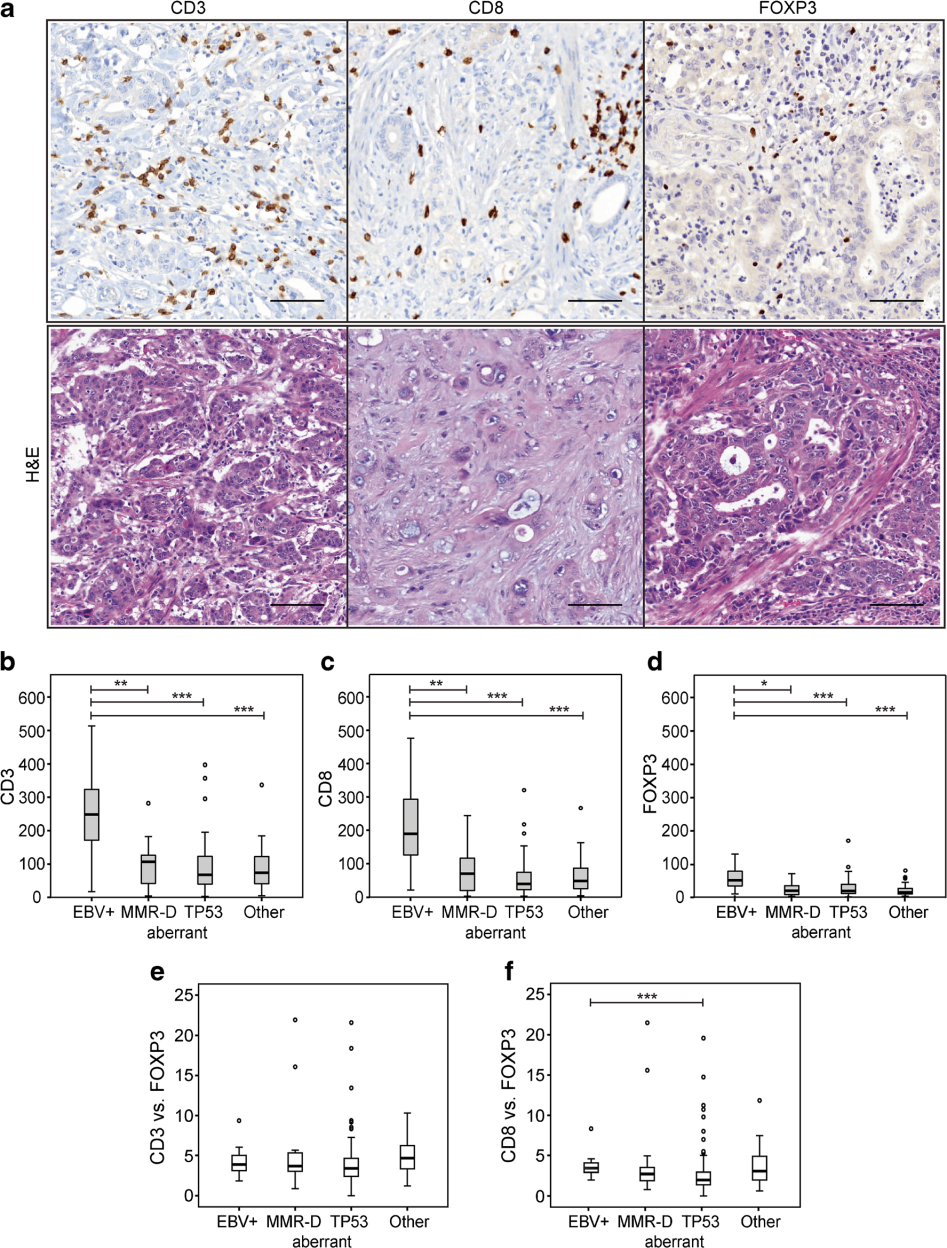
CD3, CD8, and FOXP3 T lymphocytes in four molecular subtypes of intestinal-type gastric adenocarcinoma. **a** Representative immunohistochemistry images of the different T lymphocyte subsets and corresponding hematoxylin-eosin images of gastric cancer tumor tissue (from left to right): CD3+ T lymphocytes, CD8+ cytotoxic T lymphocytes, and FOXP3+ regulatory T lymphocytes. Scale bar = 100 μm. **b**–**f** Box plot visualization of T lymphocyte subsets in the different intestinal-type gastric cancer molecular subtypes. **b** CD3+ T lymphocytes. **c**. CD8+ T lymphocytes. **d** FOXP3+ T lymphocytes. **e** CD3+/FOXP3+ lymphocytes. **f** CD8+/FOXP3+ lymphocytes. *Y*-axis = number of lymphocytes/0.2mm^*2*^. Outliers are denoted with a circle.*p = 0.002, **p = 0.001, ***p < 0.0001

The number and proportion of T lymphocyte in gastric cancer specimens was assessed using three markers: CD3, marking the entire T cell population; CD8, marking the killer/effector T cells; and FOXP3, marking the regulatory T lymphocytes (Fig. 1a). Initial analyses were made separately for the T lymphocyte infiltrates in the invasive front and central area of the tumor. As the results showed no statistical differences between the locations (data not shown), we in subsequent analyses combined results from all four TMA cores of each single tumor. CD3 could be evaluated in 180 (*n* = 98 %), CD8 in 170 (*n* = 92%), and FOXP3 in 173 (*n* = 94 %) tumors. The number of infiltrating lymphocytes varied largely between individual tumors, values ranging from 2.9 to 5140.0/0.2 mm^2^ for CD3+ cells, 3.9 to 483.8/0.2 mm^2^ for CD8+ cells, and 0.3 to 178.2/0.2 mm^2^ for FOXP3+ cells (Table 2)**.**

**Table 2:** The number of lymphocytes in intestinal-type tumors^a^ and their association (*p* value) with different molecular subtypes^b^

	*n*	Mean	Min	Median	Max		MMR-D	TP53 aberrant	Other
CD3+						CD3+			
EBV-positive	14	248.3	17.4	248.0	514.0	EB-positive	0.001	< 0.0001	< 0.0001
MMR-D	18	103.3	4.0	107.0	281.9	MMR-D		0.303	0.382
TP53 aberrant	94	87.2	2.9	68.0	397.1	TP53 aberrant			0.849
Other	51	85.5	5.3	73.7	336.8				
CD8+						CD8+			
EBV-positive	14	211.4	22.4	192.9	483.8	EBV-positive	0.001	< 0.0001	< 0.0001
MMR-D	18	82.2	4.0	72.3	248.4	MMR-D		0.096	0.339
TP53 aberrant	94	55.2	3.9	41.2	325.9	TP53 aberrant			0.279
Other	51	62.4	4.9	49.9	271.3				
FOXP3+						FOXP3+			
EBV-positive	14	61.3	9.4	54.1	137.8	EBV-positive	0.002	< 0.0001	< 0.0001
MMR-D	18	24.0	2.0	20.0	75.0	MMR-D		0.698	0.613
TP53 aberrant	95	28.2	0.3	19.0	178.2	TP53 aberrant			0.150
Other	51	21.3	2.3	15.0	80.6				

^a^Excluding one tumor with negative E-cadherin

^b^The *p* values were calculated with Mann-Whitney *U* test

A significantly higher number of infiltrating T lymphocytes was seen in the EBV-positive tumors in comparison with any other subtype (Fig. 1b–d). This finding was seen for all three T lymphocyte populations (CD3+, CD8+, and FOXP3+). Between the three other molecular subtypes, no significant differences in the number of T lymphocytes or the cytotoxic or regulatory subtypes were detected. EBV positivity was also associated with high CD8+/FOXP3+ ratio (*p* = 0.002), whereas the TP53 aberration subtype was associated with low CD8+/FOXP3+ (*p* < 0.0001) and CD3+/FOXP3+ (*p* = 0.033) ratios. The CD3+/FOXP3+ and CD8+/FOXP3+ ratio within the different molecular gastric subtypes is shown in Fig. 1e and f, respectively.

For further analyses, the tumors were dichotomized into two categories dependent on whether T lymphocyte subset values or subset ratios were above or below the median. In these analyses, EBV-positive tumors had higher levels of CD3+ cells (*p* = 0.002), CD8+ cells (*p* = 0.001), and FOXP3+ cells (*p* = 0.002) as compared with EBV-negative tumors. In addition, CD8+/FOXP3+ ratio was significantly higher in EBV-positive tumors than in EBV-negative tumors (*p* = 0.002) (Table 3). In contrast, tumors with TP53 aberration were associated with lower number of CD8+ cells (*p* = 0.02) and lower CD8+/FOXP3+ (*p* < 0.0001) and CD3+/FOXP3+ (*p* = 0.033) ratio than the TP53 wild-type tumors. MMR-D tumors did not statistically differ from TP53 aberrant or “other” subtypes, although there was a trend for elevated number of CD8+ cells (*p* = 0.096, Table 2).

**Table 3:** The association between lymphocyte counts and the different clinicopathologic variables in intestinal-type adenocarcinomas

	CD3			CD8			FOXP3			CD8/FOXP3			CD3/FOXP3		
	Below median	Above median	*p* value	Below median	Above median	*p* value	Below median	Above median	*p* value	Below median	Above median	*p* value	Below median	Above median	*p* value
EBV															
Positive	1 (1.2)	13 (13.8)	0.002^a^	1 (1.2)	13 (14.1)	0.001^a^	1 (1.2)	13 (13.4)	0.002^a^	2 (2.1)	12 (14.6)	0.002^a^	8 (7.9)	6 (7.7)	0.96
Negative	83 (98.8)	81 (86.2)		85 (98.8)	79 (85.9)		81 (98.8)	84 (86.6)		95 (97.9)	70 (85.4)		93 (92.1)	72 (92.3)	
MMR															
MMR-D	6 (7.1)	12 (12.8)	0.21	5 (5.8)	13 (14.1)	0.07	8 (9.8)	10 (10.3)	0.90	8 (8.2)	10 (12.2)	0,38	11 (10.9)	7 (9.0)	0.67
MMR-P	78 (92.9)	82 (87.2)		81 (94.2)	79 (85.9)		74 (90.2)	87 (89.7)		89 (91.8)	72 (87.8)		90 (89.1)	71 (91.0)	
TP53															
Aberration	52 (61.9)	48 (51.1)	0.15	56 (65.1)	44 (47.8)	0.02	45 (54.9)	56 (57.7)	0.70	68 (70.1)	33 (40.2)	< 0.0001	64 (63.4)	37 (47.4)	0.03
Wild-type	32 (38.1)	46 (48.9)		30 (34.9)	48 (52.2)		37 (45.1)	41 (42.3)		29 (29.9)	49 (59.8)		37 (36.6)	41 (52.6)	
E-cadherin															
Aberration	0 (0.0)	3 (3.2)	0.250^a^	0 (0.0)	3 (3.3)	0.25	1 (1.3)	2 (2.1)	< 0.999^a^	1 (1.1)	2 (2.4)	0.60	2 (2.0)	1 (1.3)	<.0.999^a^
Wild-type	81 (100.0)	91 (96.8)		83 (100.0)	89 (96.7)		78 (98.7)	95 (97.9)		93 (98.9)	80 (97.6)		96 (98.0)	77 (98.7)	
Patient sex															
Female	35 (41.2)	28 (29.2)	0.09	36 (41.4)	27 (28.7)	0.07	36 (43.9)	28 (28.0)	0.03	32 (32.3)	32 (38.6)	0.38	35 (34.3)	29 (36.3)	0.79
Male	50 (58.8)	68 (70.8)		51 (58.6)	67 (71.3)		46 (56.1)	72 (72.0)		67 (67.7)	51 (61.4)		67 (65.7)	51 (63.7)	
Age at diagnosis															
Below median	36 (42.4)	44 (45.8)	0.64	38 (43.7)	42 (44.7)	0.89	37 (45.1)	43 (43.0)	0.77	48 (48.5)	32 (38.6)	0.18	49 (48.0)	31 (38.8)	0.21
Above median	49 (57.6)	52 (54.2)		49 (56.3)	52 (55.3)		45 (54.9)	57 (57.0)		51 (51.5)	51 (61.4)		53 (52.0)	49 (61.3)	
Location															
Distal esophagus	9 (10.6)	10 (10.4)	0.20	9 (10.3)	10 (10.6)	0.29	7 (8.5)	12 (12.0)	0.03	15 (15.2)	4 (4.8)	0.12	12 (11.8)	7 (8.8)	0.88
GOJ/cardia	25 (29.4)	32 (33.3)		26 (29.9)	31 (33.0)		22 (26.8)	35 (35.0)		32 (32.3)	25 (30.1)		32 (31.4)	25 (31.3)	
Corpus	18 (21.2)	30 (31.3)		19 (21.8)	29 (30.9)		18 (22.0)	31 (31.0)		24 (24.2)	25 (30.1)		28 (27.5)	21 (26.3)	
Antrum/pylorus	33 (38.8)	24 (25.0)		33 (37.9)	24 (25.5)		35 (42.7)	22 (22.0)		28 (28.3)	29 (34.9)		30 (29.4)	27 (33.8)	
Grade															
I	12 (14.1)	5 (5.2)	0.00	13 (14.9)	4 (4.3)	0.01	10 (12.2)	7 (7.0)	0.24	11 (11.1)	6 (7.2)	0.02	9 (8.8)	8 (10.0)	0.30
II	49 (57.6)	38 (39.6)		46 (52.9)	41 (43.6)		42 (51.2)	45 (45.0)		55 (55.6)	32 (38.6)		54 (52.9)	33 (41.3)	
III	24 (28.2)	53 (55.2)		28 (32.2)	49 (52.1)		30 (36.6)	48 (48.0)		33 (33.3)	45 (54.2)		39 (38.2)	39 (48.8)	
T															
T1	8 (9.4)	11 (11.5)	0.53	10 (11.5)	9 (9.6)	0.33	5 (6.1)	14 (14.0)	0.22	11 (11.1)	8 (9.6)	0.61	11 (10.8)	8 (10.0)	0.91
T2	10 (11.8)	18 (18.8)		9 (10.3)	19 (20.2)		11 (13.4)	17 (17.0)		17 (17.2)	11 (13.3)		14 (13.7)	14 (17.5)	
T3	38 (44.7)	40 (41.7)		40 (46.0)	38 (40.4)		36 (43.9)	42 (42.0)		44 (44.4)	34 (41.0)		45 (44.1)	33 (41 3)	
T4	29 (34.1)	27 (28.1)		28 (32.2)	28 (29.8)		30 (36.6)	27 (27.0)		27 (27.3)	30 (36.1)		32 (31.4)	25 (31.3)	
Stage															
I	14 (16.5)	23 (24.0)	0.46	15 (17.2)	22 (23.4)	0.34	11 (13.4)	26 (26.0)	0.21	21 (21.2)	16 (19.3)	0.98	19 (18.6)	18 (22.5)	0.63
II	39 (45.9)	37 (38.5)		39 (44.8)	37 (39.4)		38 (46.3)	38 (38.0)		41 (41.4)	35 (42.2)		45 (44.1)	31 (38.8)	
III	26 (30.6)	32 (33.3)		26 (29.9)	32 (34.0)		28 (34.1)	31 (31.0)		32 (32.3)	27 (32.5)		31 (30.4)	28 (35.0)	
IV	6 (7.1)	4 (4.2)		7 (8.0)	3 (3.2)		5 (6.1)	5 (5.0)		5 (5.1)	5 (6.0)		7 (6.9)	3 (3.8)	

^a^*p* value calculated with Fisher’s exact test; otherwise *χ*2 test

### CD3+, CD8+, and FOXP3+ T lymphocyte subsets in relation to the clinicopathological characteristics

Additional comparisons were carried out to find associations between the dichotomized T lymphocyte subset or subset ratio groups and clinicopathological features. Results on these associations are presented in Table 3.

Above median values of CD3+ or CD8+ T lymphocytes were significantly associated with poor differentiation (*p* = 0.001 for CD3+, *p* = 0.005 for CD8+ and *p* = 0.018 for CD8/FOXP3 ratio). Tumor localization was associated with the presence of FOXP3+ cells (*p* = 0.029), low numbers of FOXP3+ cells being more common in tumors of the antrum/pylorus. Male patients had more often tumors with high levels of FOXP3+ lymphocytes (*p* = 0.025). No significant association could be detected between the frequency of CD3+, CD8+, or FOXP3+ T cells and patient age, tumor size, or tumor location.

### CD3+, CD8+, and FOXP3+ cells and other clinical features in relation to survival

**Fig. 2:**
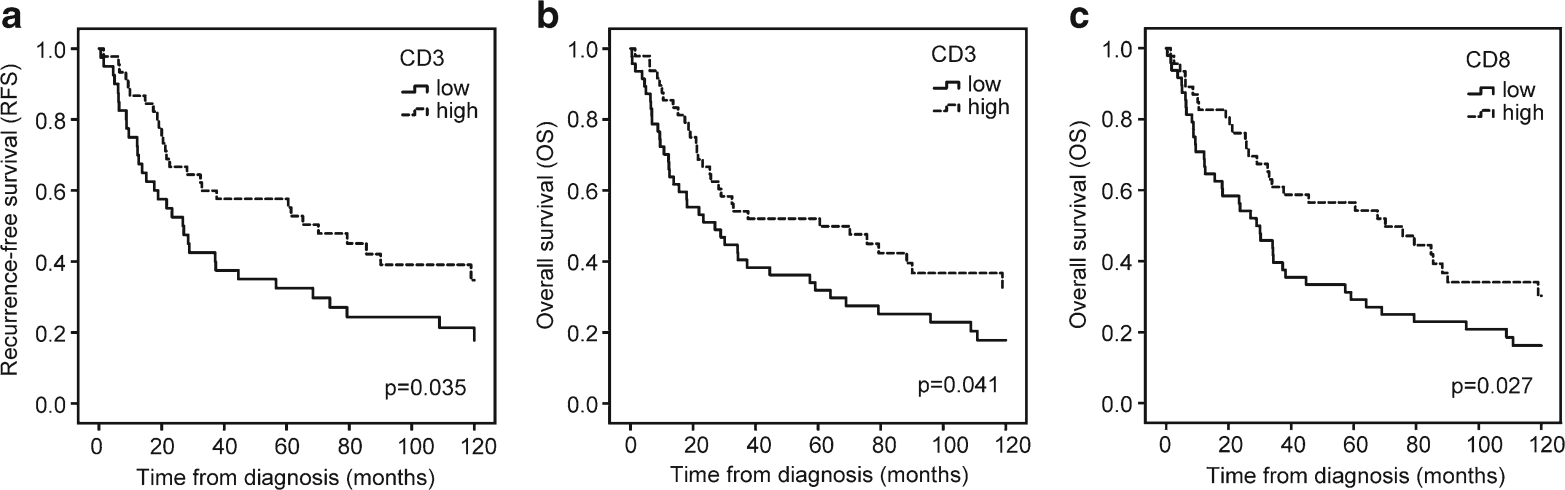
Kaplan-Meier analysis for recurrence-free survival and overall survival of patients with intestinal-type gastric cancer in relation to T lymphocyte infiltration. **a** Recurrence-free survival (CD3). **b** and **c** Overall survival (CD3 and CD8, respectively)

For survival analyses, the gastric cancer tumors were divided into four quartiles depending on the number CD3+, CD8+, and FOXP3+ T lymphocytes. High number of CD3+ cells was significantly associated with longer recurrence-free survival (log-rank test, *p* = 0.035, Fig. 2a). Patients with tumors in the highest quartile of CD3+ (log-rank test, *p* = 0.041) and CD8+ (log-rank test, *p* = 0.027) cells also had significantly higher overall survival than patients with tumors in the lowest quartile of CD3+ and CD8+ cells (Fig. 2b and c, respectively).

In univariate analysis, the highest and lowest quartiles of CD3+, CD8+, and FOXP3+ were associated with RFS and OS. Additional parameters in this comparison were MMR status, tumor size, and stage (Table 4). High amount of CD3+ cells associated with longer RFS (HR 0.58, 95% CI: 0.35–0.97) and OS (HR 0.61, 95% CI 0.38–0.98). High amount of CD8+ cells was associated with OS (HR 0.59, 95% CI: 0.37–0.95) but not with RFS. The amount of FOXP3+ cells did not associate with survival. Patients with MMR-D tumors had longer OS than patients with MMR-proficient (MMR-P) tumors. As expected, large tumor size and advanced stage correlated with shorter RFS and OS. The results are shown in Table 4.

**Table 4:** Recurrence-free survival (RFS) and overall survival (OS) of patients with intestinal-type gastric adenocarcinoma

	Univariate survival analysis for RFS						Univariate survival analysis for OS					
	Number of patients	RFS, median (months)	*p* value (log-rank test)	*p* value (Cox’s test)	HR	95% CI	Number of patients	OS, median (months)	*p* value (log-rank test)	*p* value (Cox’s test)	HR	95% CI
MMR status												
MMR-P	138	32.6	0.067	0.072	0.53	0.27–1.06	161	28.7	0.030	0.033	0.48	0.24–0.94
MMR-D	17	93.0					17	93.0				
CD3												
Q1	40	26.8	0.036	0.038	0.58	0.35–0.97	47	27.0	0.041	0.043	0.61	0.38–0.98
Q3	45	70.1					48	60.5				
CD8												
Q1	38	28.9	0.065	0.067	0.62	0.37–1.04	48	28.9	0.027	0.028	0.59	0.37–0.95
Q3	43	70.1					46	70.1				
FOXP3												
Q1	34	26.8	0.050	0.053	0.61	0.37–1.01	36	34.2	0.247	0.249	0.75	0.47–1.22
Q4	50	65.2					56	43.4				
T												
T1	17	65.2	0.025	0.016	1.1	1.05–1.58	19	32.7	0.005	0.007	1.1	1.08–1.57
T2	26	79.3					28	70.1				
T3	69	30.0					77	30.2				
T4	47	27.6					58	22.9				
Stage												
I	36	70.1	0.042	0.012	1.1	1.07–1.75	39	70.1	< 0.0001	< 0.0001	1.1	1.24–1.91
II	72	32.6					75	33.8				
III	51	22.6					58	22.6				
IV							10	1.9				

In multivariate analysis, high amount of CD3+ cells remained as an independent prognostic factor for longer RFS (HR 0.57, 95% CI: 0.34–0.96) and OS (HR 00.03, 95% CI: 00.003–0.31) and high amount of CD8+ cells for OS (HR 17.2, 95% CI: 1.78–1660.0) (Table 5). Advanced stage remained as an independent prognostic factor for OS (HR 8.25, 95% CI: 2.40–28.3). EBV-positive cancers are associated with improved survival as compared with other gastric cancer subtypes [22]. Thus, in our cohort, the association between CD3+ and CD8+ high tumors and longer RFS and OS could be explained by overrepresentation of EBV-positive tumors in the highest quartile of CD3 and CD8+ cancers. However, when including only the MMR-D, TP53 aberrant, and “other” subtypes, the amount of CD8+ T cells remained significantly associated with OS in univariate (log-rank test, *p* = 0.043; Cox test, *p* = 0.046; HR 0.58; 95% CI: 0.34–0.99) and in multivariate analysis (Cox test, *p* = 0.039; HR 0.67; 95% CI: 0.46–0.98).

**Table 5:** Multivariate analysis of recurrence-free survival (RFS) and overall survival (OS) of patients with intestinal-type gastric adenocarcinoma

	Number of patients	*p* value (Cox’s test)	HR	95% CI		Number of patients	*p* value (Cox’s test)	HR	95% CI
MMR status					MMR status				
MMR-P (ref)					MMR-P (ref)	75	0.102	0.37	0.11–1.22
MMR-D					MMR-D	7			
CD3					CD3				
Q1 (ref)	49	0.035	0.57	0.34–0.96	Q1 (ref)	44	0.003	0.03	0.003–0.31
Q3	48				Q3	38			
CD8					CD8				
Q1 (ref)					Q1 (ref)	43	0.014	17.2	1.78–166.0
Q3					Q3	39			
Stage					Stage				
I (ref)	21				I (ref)	17			
II	42	0.960	0.98	0.51–1.91	II	31	0.391	1.1	0.66–2.92
III	34	0.503	1.1	0.64–2.51	III	29	0.866	1.7	0.48–2.37
IV					IV	5	0.001	1.8	2.40–28.3

## Discussion

Host immune response towards cancer varies between cancer types and individuals. The amount of tumor-infiltrating T lymphocytes has prognostic implications, and may indicate response to immune modulating therapy. While recent studies have shown that intestinal-type gastric cancer consists of several molecular subtypes, the potential of the different subtypes to elicit host immune response is not well-known. Here, we analyzed the presence of intratumoral of CD3+, CD8+, and FOXP3+ T lymphocytes in intestinal gastric cancers representing four different molecular subtypes (EBV, MMR-D, TP53 aberrant, and “others”). Our results show that the EBV+ cancers differ from all other subtypes by increased lymphocyte infiltration. While the other subtypes did not differ in the absolute number of T lymphocyte, there was a large variation between individual tumors. Interestingly, the ratio of FOXP3+ Treg vs. CD3+ or CD8+ cells was highest in the TP53 aberrant subtype, which also has the worst prognosis. The presence of CD3+ and CD8+ T lymphocytes associated with survival in the entire cohort, and in the subset from which the EBV+ tumors were excluded.

The association between a high number of T lymphocytes and EBV positivity has been previously reported. Ma et al. found that in a cohort of 16, MMR-D, 7 EBV+, and 21 non-EBV/MMR-P tumors, MMR-D, and EBV+ subtypes harbored two-fold higher densities of CD8+ T lymphocytes in the invasive part of the tumors [23]. In another set of 20 EBV+ and 28 EBV-gastric cancers, Van Beek et al. reported high density of intratumoral lymphocytes, high density of activated granzyme B expressing CD8+ T lymphocytes and a CD8/CD4 ratio > 1 in the EBV+ subtype [24]. These studies included relatively small sample numbers from patients of Asian origin. Here, we confirm these results in a larger sample set of intestinal gastric cancers of Caucasian origin.

Previous studies have indicated that MMR-D tumors harbor an increased numbers of T cells. These findings are mostly shown in colorectal and endometrial cancer types, but similar correlation has also been suggested for gastric cancer [25–27]. In our material, the MMR-D tumors did not significantly differ in number of CD3+, CD8+, or FOXP3+ cells from TP53 aberrant and “other” subtypes, although there was a trend towards increased number of CD8+ cells. It is unclear why our results on intestinal-type gastric cancer do not recapitulate the findings from other cancer types. One reason may be the relatively small size of distinct subtypes. It should, however, be noted that results regarding this topic are sparse, and the published gastric cancer studies on the correlation between MMR-D tumors and T lymphocyte counts either lack statistical analyses [28], or do not show increased number of T lymphocytes [29]. An exception is the study by Xing et al., which shows an increased number of CD3+ cells but not CD8+ cells in MMR-D tumors as compared with MMR-P tumors [30]. To conclude, more studies are needed to find out whether MMR-D intestinal gastric cancers have more T lymphocytes than the TP53 aberrant and “other” subtypes.”

It has been suggested that cancer cells within MMR-D subtype are able to attract mainly cytotoxic CD8+ T lymphocytes [31]. Recently, immune checkpoint inhibitors have emerged as a new treatment option for certain immunogenic gastric cancers. However, not all patients with T lymphocyte–inflamed tumors respond to the treatment, indicating that the high number of T lymphocytes is not the only predictor of treatment outcome for MMR-D subtypes but also the complex crosstalk between cancer cells, immune cells, and the tumor microenvironment should be considered [31, 32].

We noticed a decreased ratio of CD3+/FOXP3+ and CD8+/FOXP3+ cells in the TP53 aberrant tumors when compared with any of the other subtypes. A reduced CD8+/FOXP3+ ratio has been associated with aggressive non-luminal tumors in breast cancer [33]. In gastric cancer, this is a novel finding and could partly explain the worse prognosis associated with the TP53 subtype. The mutation and chromosomal instability in TP53 subtype in gastric cancer has been suggested as an important biological mechanism driving immunosuppression in the TP53 subtypes [34].

Survival analyses showed a positive association between the amount of CD3+ and CD8+ T cells and increased overall survival. This finding remained significant even when the EBV+ subtype with the highest number of T cells was excluded. This indicates a variation in the host immune response between individuals and a potential prognostic value for T lymphocyte infiltration in gastric cancer. High expression of CD8+ T lymphocytes had the strongest prognostic association across all four gastric cancer subtypes. Dense intratumoral infiltration of CD3+ T lymphocytes was also significantly associated with longer RFS of the patients, which remained as an independent prognostic factor for longer overall survival in a multivariate analysis. This is in line with a study by Lee et al., where CD3+ is reported as an independent favorable prognostic factor in gastric cancer [35].

Our study is utilizing a European cohort to investigate the numbers of TILs within four molecular subtypes of intestinal-type gastric cancer. The previous studies have mostly included patients of Asian origin, with somewhat different environmental, lifestyle, and genetic risk factors. Therefore, our study is an important addition to the current knowledge of T lymphocyte infiltration in intestinal gastric cancer and its prognostic significance.

We conclude that CD3+ and CD8+ intratumoral T lymphocytes are independent prognostic markers, and among all intestinal-type gastric tumors, the EBV+ subtype is the most immunogenic molecular subtype. Better understanding of the role of tumor microenvironment and lymphocyte infiltration in immunogenic gastric cancers could support the treatment decisions when considering immunological therapies for gastric cancer patients.
